# The Natural History of Uterine Leiomyomas: Light and Electron Microscopic Studies of Fibroid Phases, Interstitial Ischemia, Inanosis, and Reclamation

**DOI:** 10.1155/2013/528376

**Published:** 2013-11-21

**Authors:** Gordon P. Flake, Alicia B. Moore, Deloris Sutton, Grace E. Kissling, John Horton, Benita Wicker, David Walmer, Stanley J. Robboy, Darlene Dixon

**Affiliations:** ^1^Cellular and Molecular Pathology Branch, National Toxicology Program (NTP), National Institute of Environmental Health Sciences (NIEHS), National Institutes of Health (NIH), Department of Health and Human Services, Research Triangle Park, NC 27709, USA; ^2^Molecular Pathogenesis Group, National Toxicology Program Laboratory (NTPL), National Toxicology Program (NTP), National Institute of Environmental Health Sciences (NIEHS), National Institutes of Health (NIH), Department of Health and Human Services, Research Triangle Park, NC 27709, USA; ^3^Biostatistics Branch, National Toxicology Program (NTP), Division of Intramural Research, National Institute of Environmental Health Sciences (NIEHS), National Institutes of Health (NIH), Department of Health and Human Services, Research Triangle Park, NC 27709, USA; ^4^Duke University Medical Center, Durham, NC 27710, USA

## Abstract

We propose, and offer evidence to support, the concept that many uterine leiomyomas pursue a self-limited life cycle. This cycle can be arbitrarily divided on the basis of morphologic assessment of the collagen content into 4 phases: (1) proliferation, (2) proliferation and synthesis of collagen, (3) proliferation, synthesis of collagen, and early senescence, and (4) involution. Involution occurs as a result of both vascular and interstitial ischemia. Interstitial ischemia is the consequence of the excessive elaboration of collagen, resulting in reduced microvascular density, increased distance between myocytes and capillaries, nutritional deprivation, and myocyte atrophy. The end stage of this process is an involuted tumor with a predominance of collagen, little to no proliferative activity, myocyte atrophy, and myocyte cell death. Since many of the dying cells exhibit light microscopic and ultrastructural features that appear distinct from either necrosis or apoptosis, we refer to this process as inanosis, because it appears that nutritional deprivation, or inanition, is the underlying cause of cell death. The disposal of myocytes dying by inanosis also differs in that there is no phagocytic reaction, but rather an apparent dissolution of the cell, which might be viewed as a process of reclamation as the molecular contents are reclaimed and recycled.

## 1. Introduction

The etiology of uterine leiomyomas (or fibroids) is unknown, and their pathogenesis has been incompletely determined. Because they are so common (80% in African-American women and 70% in Caucasian women in one study [1]), it would be reasonable to assume that women share a common risk factor for the development of fibroids. One such factor is menstruation, and perhaps more importantly is the occurrence of dysmenorrhea with associated abnormal uterine contractions [2, 3], which is estimated to occur in up to 70% of women by the fifth year after menarche [4]. Patients with primary dysmenorrhea experience varied patterns of myometrial hyperactivity, including contractions of increased amplitude, very frequent contractions, and/or a high basal tone, and this increased contractile activity is associated with a reduction in uterine blood flow [5]. If focal injury (ischemic or otherwise) to the myometrium occurs during menstruation, the reparative response could be similar to that which occurs following injury to blood vessels. In response to vascular intimal injury, smooth muscle cells of the media migrate into the intima, proliferate, and synthesize extracellular matrix [6]. During the healing response, these smooth muscle cells are thus transformed into cells that have the capacity to divide and to synthesize extracellular proteins, while losing the capacity to contract. These changes are mirrored by the electron microscopic changes in which the smooth muscle cells exhibit a decrease in contractile filaments and an increase in protein synthesizing organelles such as the rough endoplasmic reticulum, free ribosomes, and Golgi apparatus [7].

The histologic changes that characterize the smooth muscle cell response to vascular injury are similar to those that occur in uterine fibroids. Uterine fibroids are characterized by two primary histologic features: the proliferation of smooth muscle cells and the production of a collagenous matrix. While the mitotic activity of fibroids is variable and generally modest, the proliferative rate of many fibroids is greater than that of the adjacent myometrium [8]. The collagenous component of fibroids is also variable in quantity. For example, one subtype of leiomyoma, the cellular leiomyoma, usually displays little extracellular matrix, consisting primarily of closely packed fascicles of smooth muscle cells, while many fibroids contain abundant fibrous matrix, which may even exceed the smooth muscle component itself. Likewise, the size of fibroids is also quite variable, from those that are barely visible (1-2 mm) to those as large as 10 cm or more. While recognizing this heterogeneity of size, proliferative activity, cellularity, and fibrotic stroma among fibroids, it is our impression that the majority of fibroids fall between the extremes of the hypercellular tumors and the hypocellular, predominantly fibrotic tumors. And thus, it would seem that the size and growth of fibroids are probably dependent upon both the proliferation of the smooth muscle cells and the synthesis and deposition of extracellular matrix.

It has been our observation that fibroids with extensive accumulation of collagen generally exhibit less mitotic activity. Based upon this observation and the conjectural analogy of fibroid development to the reparative response of smooth muscle cells in vascular injury, we hypothesized that the growth of uterine fibroids begins with a predominantly proliferative phase that either precedes or occurs concomitantly with the production of extracellular matrix. At some stage in the life of a fibroid, however, this progressive elaboration of matrix then seems to predominate over the proliferative response, resulting in the ultimate appearance of a hyalinized, involuted tumor. With this concept in mind, we have arbitrarily divided the development of fibroids into 4 hypothetical phases based upon the quantity of collagen present in the tumor. We have further hypothesized that the proliferative rate of the tumors would be the greatest in the early phases and would then diminish with progression to the final phase of involution.

## 2. Materials and Methods

### 2.1. Study Participants and Specimens

#### 2.1.1. Study Participants

Fibroid tumor specimens were obtained by consent from women undergoing hysterectomy or myomectomy at the George Washington University Medical Center between June, 1996 and April, 1999, as part of the NIEHS Uterine Fibroid Study. Details of patient recruitment, demographics, and collection of gross pathology data have been previously reported [9].

#### 2.1.2. Specimens

Following fixation in 10% buffered formalin, representative sections of at least one and as many as six fibroid tumors were submitted from each patient. Up to six sections were taken from tumors that were of sufficient size, with four of these sections taken from the periphery of the tumor and two from the central portion of the tumor. Sections of myometrium and endometrium were also submitted from many of the patients. The fixed tissues were routinely processed, embedded in paraffin, sectioned at 4-5 *μ*m, and stained with hematoxylin and eosin. Representative sections were stained with Masson's trichrome stain to accentuate the collagen.

### 2.2. Light Microscopy

A total of 2151 sections from 460 fibroid tumors were available for microscopic examination. The slides were examined with an Olympus BX50 microscope, using objectives ranging from 2x to 100x. An Olympus ocular micrometer WHN10x-H/22 was used for nuclear measurements. The magnifications cited in the figure legends represent the original objective magnification multiplied by the tube length magnification of 3.3.

### 2.3. Developmental Phases of Fibroids

On the basis of microscopic estimation of the area of the tumor occupied by extracellular matrix in H&E stained slides, we arbitrarily categorized the fibroid tumors into four phases as follows:

 Phase 1 = no, or insignificant, collagen matrix
 Phase 2 = <10% collagen
 Phase 3 = 10–50% collagen
 Phase 4 = >50% collagen.

When more than 1 section of the tumor was available, all sections were included in the estimation of percent collagen (Table 1).

### 2.4. Mitotic Counts

The H&E stained sections of all tumors were scanned with the 20x objective of an Olympus BX50 microscope until a mitosis was identified, and then the number of mitoses in 10 high power (40x objective) fields was counted. Only structures consistent with the prometaphase, metaphase, anaphase, or telophase stages of mitosis were counted. When more than one section of a tumor was available, the mitotic counts from each section were added and the sum was divided by the number of sections to give an average mitotic count for each tumor.

### 2.5. Gross Tumor Size

Gross tumor size data (<2 cm or ≥2 cm) were available for most (86%) of the tumors examined microscopically. Following assignment of Phase (1–4) for each tumor, the gross tumor sizes were obtained from the surgical pathology records for comparison of tumor size with tumor phase.

### 2.6. Transmission Electron Microscopy (TEM)

Specimens for TEM were collected, with no identifiers or links to patient identification in accordance with guidelines by the National Institutes of Health Office of Human Subjects Research, at the time of hysterectomy or myomectomy at Duke University Medical Center in Durham, North Carolina. This on-site collection allowed for the immediate sectioning of tissue into 1 mm cubes and fixation in 2.5% glutaraldehyde. Following fixation, the tissues were processed routinely for TEM in a Lynx Automatic Tissue Processor (Electron Microscopy Sciences, Hatfield, PA) by buffer rinsing, postfixing in 1% osmium tetroxide, dehydrating in an ascending graded series of ethanols and acetone, and infiltrating and embedding in epoxy resin. Semithin sections (or “thick sections”, a 500 to 800 nm section) were cut and stained with toluidine blue, examined by a pathologist, and areas within the stained section chosen for thin sectioning (a 90 nm or “gold section”). Areas selected for thin sectioning were trimmed and cut and then placed on copper grids. The grids were examined on a Tecnai G2 12 BioTwin 120 KV TEM (FEI, Hillsboro, Oregon). Digital photomicrographs were taken from selected areas of interest.

### 2.7. Statistical Analysis of Mitotic Counts

Descriptive statistics included means, standard errors of the mean, and minimum and maximum mitotic counts per tumor. Because mitotic counts were not normally distributed, counts were compared across phases using one-sided Mann-Whitney tests.

## 3. Results

### 3.1. Developmental Evolution of Fibroids: Phasing by Collagen Content

**Figure 1:**
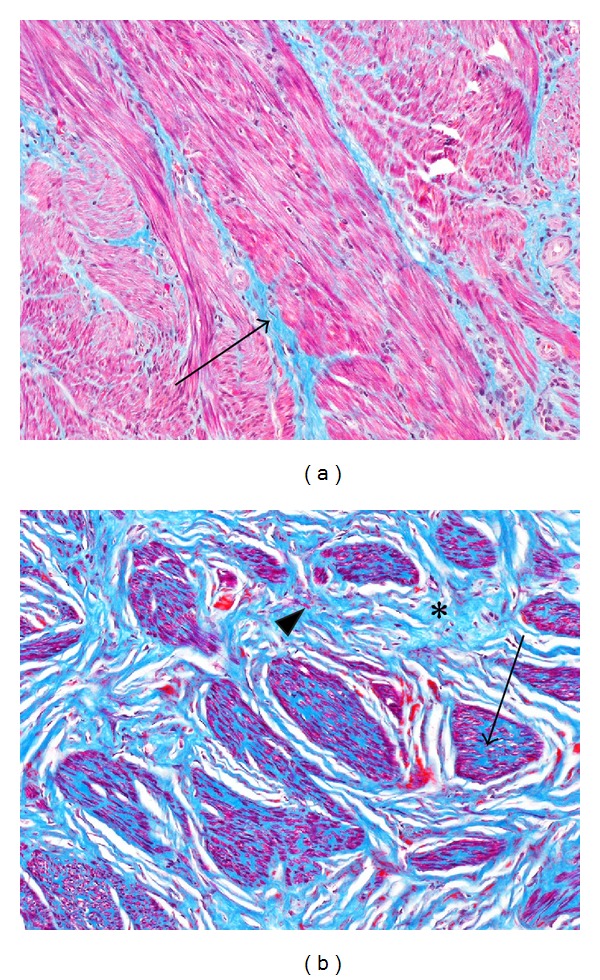
Intrafascicular and interfascicular fibrosis. (a) Masson's trichrome stain of normal myometrium for comparison with (b) Phase 3 fibroid, also stained with Masson's trichrome, which stains muscle red and collagen blue. Note that in the myometrium (a) most of the blue staining collagen is between the fascicles (interfascicular) (arrow), whereas blue collagenous stroma in the fibroid is also noted between the individual myocytes of the muscle fascicles (intrafascicular fibrosis) (arrow), without the interposition of fibroblasts, indicating that the collagenous stroma is being produced by the myocytes themselves. In addition, the interfascicular blue collagenous stroma (asterisk) is more abundant in the fibroid (b) than in the myometrium (a). Red staining cells within the interfascicular stroma of the fibroid (arrowhead) probably represent transformed myocytes, or fibroblasts. Original objective magnification of (a) and (b): 10x.

Phasing of fibroids was based upon the microscopically estimated percent of total tumor area occupied by extracellular fibrous matrix in H&E stained slides. Small amounts of collagenous matrix around blood vessels and between muscle fascicles, unaccompanied by obvious spindle cell or fibroblastic proliferation, were considered to be part of the normal structural support and were not included in the total percentage. This perivascular and interfascicular matrix appeared to be a relatively minor component in most fibroids except for those tumors assigned to the Phase 1 category in which fibrous matrix was otherwise minimal. Collagen deposition within the tumors was variably interfascicular, intrafascicular, or a combination of the two (Figure 1). After applying the criteria discussed in the methods section in which tumors were categorized on the basis of percent collagen content (Phase 1 = no, or insignificant, collagen matrix; Phase 2 = <10% collagen; Phase 3 = 10–50% collagen; Phase 4 = >50% collagen), it became clear that most fibroids would fall into the Phase 3 category, lesser numbers into the Phase 2 category, and the least number into the Phase 1 and 4 categories (Table 2). Although most fibroid tumors exhibited collagen production, there were a few tumors that showed no obvious fibrosis; these were generally small and may significantly represent an early stage of development, and for this reason, the category of Phase 1 was created. Among patients with more than one fibroid, the majority (69%) were found to have tumors in more than one phase, suggesting that the tumors in these patients either arose at different times or were genetically different. 

**Figure 2:**
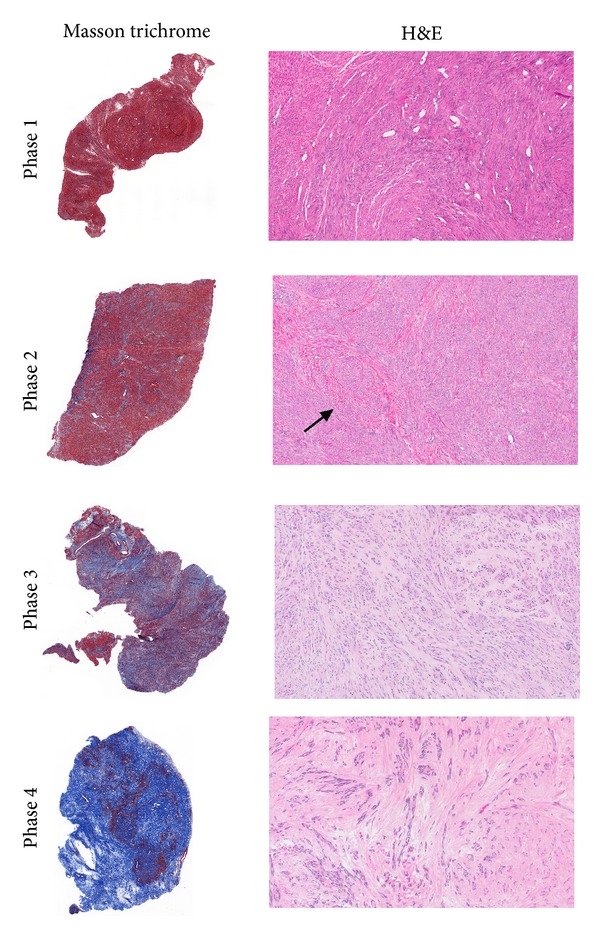
Fibroid Phases 1–4. Representative examples of the four phases of fibroid development are shown, with the Masson trichrome stain (1x image) on the left and the H&E stain of the same tumor (10x image) on the right. The progressive increase in blue staining collagen from Phase 1 to Phase 4 is well shown in the Masson trichrome stained sections. The corresponding H&E images on the right also demonstrate the virtual absence of collagen in the Phase 1 tumor, the appearance of interspersed pink collagenous fibers (arrow) in Phase 2, the more abundant pale pink collagenous stroma of Phase 3, and the predominance of pink, hyalinized stroma in Phase 4. Note also the abundance of microvessels (small ovoid spaces) in Phase 1 and the paucity of vessels in Phases 3 and 4.

Phase 1 and Phase 2 tumors consist predominately of myocytes, whereas in Phase 3 and Phase 4 tumors, the balance is progressively tilted towards a predominance of dense extracellular matrix (Table 1). As the quantity of collagenous tissue increases, the microvessel density often appears to decrease; this is particularly noticeable in comparing Phase 1 and Phase 4 tumors. Representative examples of H&E and Masson trichrome stained fibroids from each of the 4 Phases are shown in Figure 2.

Mitotic figures were infrequent in most tumors and were not identified in the Phase 4 tumors (Table 2). Phase 3 tumors also showed a decline in average mitotic count, compared to Phases 1 and 2, consistent with the hypothesis of decreased proliferation as the collagenous matrix accumulates in the later developmental phases. The differences in average mitotic count between Phases 1 and 4 (*P* = 0.0003), Phases 2 and 3 (*P* = 0.0105), Phases 2 and 4 (*P* < 0.0001), and Phases 3 and 4 (*P* < 0.0001) were all statistically significant. Myometrial samples were also available from 67 of the patients. Out of the total of 287 myometrial samples, mitotic activity (1/10 HPF) was found in only one sample from each of two patients.

Analysis of gross tumor size data revealed an increase in average size of tumors in Phases 3 and 4, as compared to Phases 1 and 2 (Table 3). The trend towards increased tumor size with progression in phase is statistically significant, with a one-sided Cochran-Armitage trend value of *P* = 0.0001. This finding correlates with our hypothesis that the growth of the tumors is the result of both the proliferation of myocytes and the accumulation of extracellular collagen. In addition, this result, in combination with the reduction in mitotic count in Phases 3 and 4, further supports our hypothesis that as the tumors grow and accumulate collagen, the proliferative rate of the myocytes eventually declines.

### 3.2. Phases 1 and 2: Proliferation and Synthesis of Collagen (Phenotypic Transformation)

**Figure 3:**
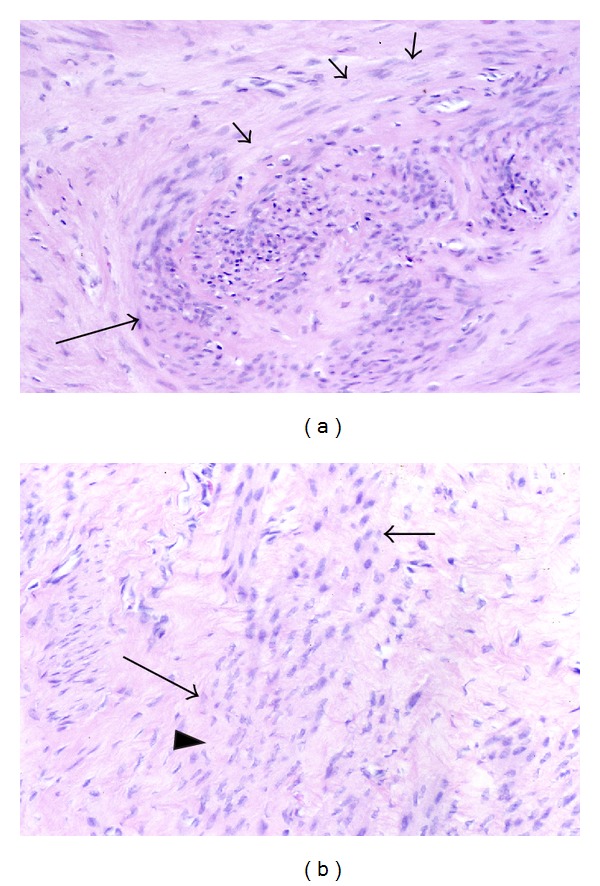
Phenotypic transformation from smooth muscle cells to fibroblast-like cells. In (a), the cells in the center and to the right are in close fascicular apposition and exhibit the typical pink cytoplasm of smooth muscle cells, while the cells above and to the left are more widely separated and have a paler cytoplasm resembling that of fibroblasts. Note that as the smooth muscle cells at the bottom (long arrow) appear to stream upwards and then to the right, there is a morphologic transformation to fibroblast-like cells with paler cytoplasm (short arrows). A similar transformation is noted in (b) as the organized, closely packed smooth muscle cells with pinker cytoplasm at the top (short arrow) transform into more widely spaced, paler fibroblast-like cells in the bottom half (long arrow). Note that the transformed cells have already produced a collagenous stroma (arrowhead), and that the cytoplasm of the transformed cells is less distinct and not clearly contiguous with that of their neighbors. Original magnification of (a) and (b): 66x.

At the interface between fascicles of myocytes and zones of collagenous matrix in leiomyomas, the myocytes often exhibit cytologic changes that are evident with the light microscope. Chief among these are the reduction of eosinophilic cytoplasm and the tendency of nuclei to become thinner and more pointed at the poles. The cytoplasmic pallor of these transformed myocytes is suggestive of a diminution in the number of myofilaments, which occupy most of the cytoplasm of normal smooth muscle cells (Figure 3). In addition, the nuclei of the transformed cells sometimes appear to be more widely spaced because of the intervening stroma between the cells.

**Figure 4:**
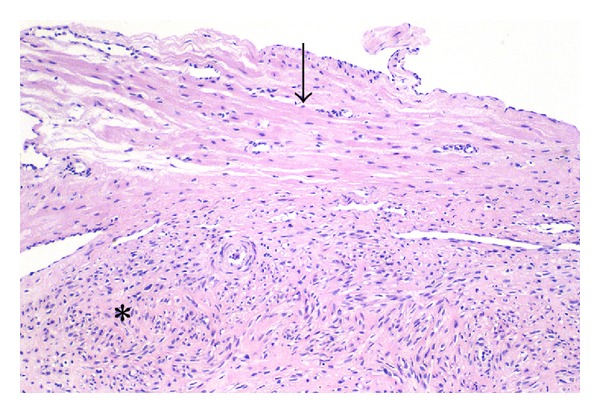
Phenotypic transformation: loss of parallel orientation of fibers. The parallel, linear orientation of the myometrial smooth muscle cells (arrow) contrasts with the haphazard, disorganized arrangement of the fibroid tumor cells (asterisk). Original magnification: 33x.

If the cytoarchitectural features of leiomyomas are compared to the myometrium itself, several differences are apparent. First, it is evident that the fascicular pattern of the myometrium becomes less well defined in leiomyomas, sometimes appearing microfascicular, sometimes disorganized and haphazardly fascicular, and sometimes relatively patternless. In addition, the parallel linear arrangement of normal myocytes usually noted in the myometrium is also replaced to varying degrees by a less orderly and sometimes completely disorganized pattern of cells in fibroid tumors (Figure 4). Since the muscle fascicle is important to coordinated contraction and might be considered the functional unit of the myometrium, the loss of this pattern signals an important deviation from the normal contractile phenotype.

**Figure 5:**
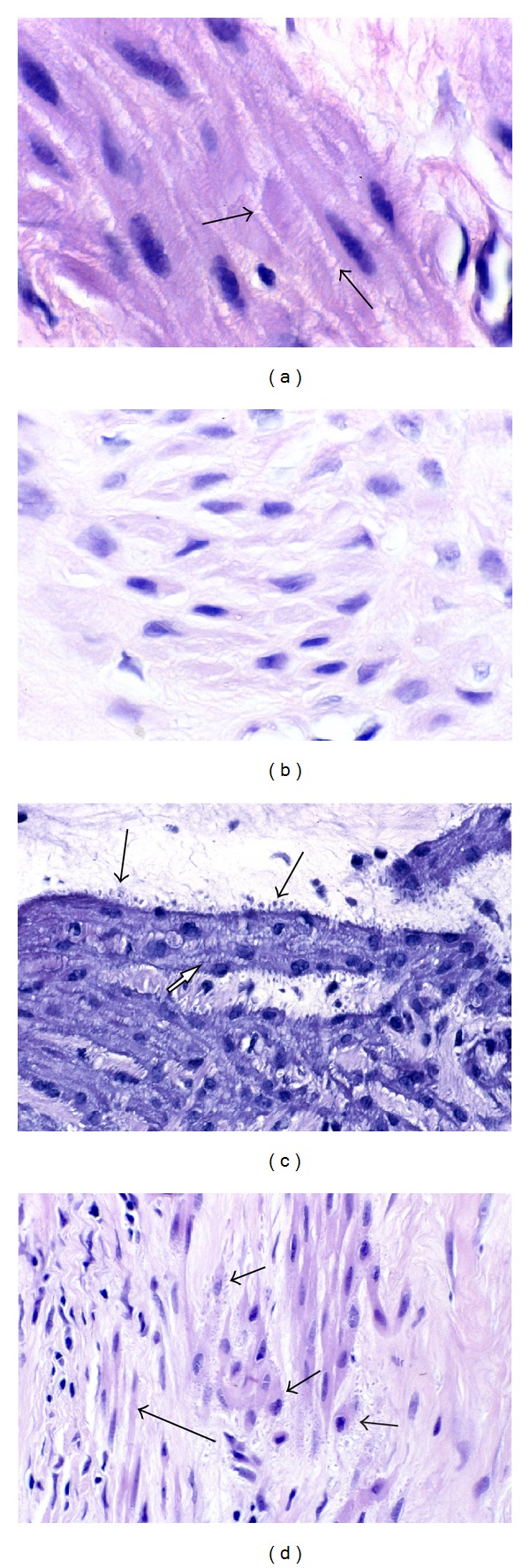
Phenotypic transformation: loss of lateral bars. Lateral bars (arrows) connecting myometrial smooth muscle cells in (a) are believed to represent sites of gap junction attachments between adjacent cells for purposes of coordinated contraction. These lateral bars, or intercellular attachments, are not seen between the fibroid tumor cells in (b). We believe that this is another indication of the phenotypic transformation to non-contractile cells. In (c), many of the fibroid myocytes exhibit lateral buds (long arrows), imparting a knobby appearance to the free borders of the cells. It is thought that these buds may represent remnants of the lateral bars. Note that the cytoplasmic borders of contiguous myocytes within the fascicle, however, do not show these lateral buds, but rather show retention of lateral bars, or attachments (short arrow), as noted in the myometrium. In (d), there are a few myocytes displaying lateral buds, which have a more frayed or feathered appearance (short arrows). Note that in the fully transformed cells on the left (long arrow), there are neither lateral bars or lateral buds. Original magnification of (a) and (b): 330x, (c) and (d): 132x.

In some sections of myometrium, close examination of myometrial myocytes with the 40x or 100x objectives reveals lateral intercellular attachments between adjacent myocytes (Figure 5(a)). We refer to these as lateral bars, and it is significant that these often disappear as myocytes undergo transformation in leiomyomas. We believe that these lateral bars are the result of 2 phenomena: (1) disproportionate shrinkage of myocytes, in comparison to the interstitial (intercellular) tissue during the alcohol dehydration step of tissue processing and (2) the tenacity of gap junctions connecting adjacent myocytes. Myocytes shrink disproportionately during the dehydration phase of tissue processing because of the high water content of muscle cells. Despite the retraction of muscle cells from each other, however, the remarkable adherence of the gap junctions remains unbroken, resulting in lateral linear extensions of the myocyte cytoplasm. Since the gap junctions are critical conduits for the influx of calcium during coordinated contraction, the loss of these lateral bars in leiomyomas is further indication of transformation to a phenotype in which contraction is no longer important (Figure 5(b)). The lateral bars also attest to the plasticity of the myocytes, a property which would be expected in a contractile cell and which is probably lost as the cells undergo transformation and reduce their content of actin filaments. Myocytes with short, stubby lateral buds are sometimes seen and are thought to represent a transitional, intermediate stage in the transformation process, as the myocytes retract from their neighbors en route to assuming their proliferative, synthesizing phenotype (Figures 5(c) and 5(d)). Interestingly, these lateral bars are not seen in electron micrographs of myometrium, which we believe is related to the postfixation step with osmium tetroxide that stabilizes proteins and causes cell swelling that tends to offset the shrinking effect of the dehydration solvents [10].

**Figure 6:**
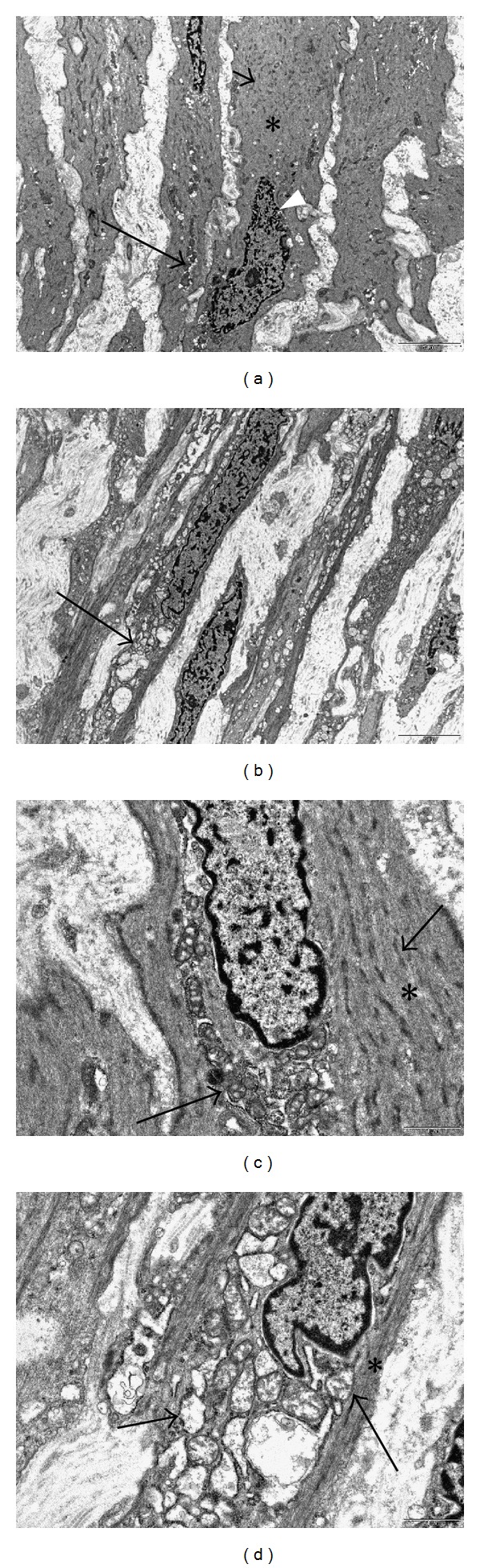
Ultrastructural features of phenotypic transformation. This side-by-side comparison of myometrial smooth muscle cells in (a) with the transformed fibroid myocytes in (b) illustrates some of the electron microscopic features of the phenotypic transformation. The myometrial myocytes in (a) display abundant, relatively homogeneous cytoplasm that is filled with fine actin filaments (asterisk), oriented in parallel array in the long axis of the cells. Also evident are interspersed dense bodies (short arrow), focal clusters of mitochondria (long arrow), and a nucleus with a crenulated nuclear border (arrowhead) that is suggestive of the contractile state. In contrast, the fibroid in (b) exhibits more intercellular stroma and slender, streamlined cells with less cytoplasm, marked reduction in the fine actin filaments, increased swollen endoplasmic reticulum (arrow), and elongated, pointed nuclei. In (c), the myometrial myocyte displays a cytoplasm filled with actin fine filaments (asterisk), interspersed dense bodies (short arrow), clusters of mitochondria (long arrow), and a nucleus with a rounded end. In contrast, the fibroid myocyte in (d) exhibits extensive, dilated endoplasmic reticulum (short arrow), swollen mitochondria (long arrow), greatly reduced myofilaments limited to the periphery of the cell (asterisk), and a nucleus with a pointed end. Original magnification of both (a) and (b): 2550x, (c) and (d): 11,500x.

By transmission electron microscopy, it is apparent, in side-by-side comparisons, that the cytoplasm of myometrial myocytes is more uniformly electron dense than the cytoplasm of leiomyoma tumor cells due to the greater concentration of thin myofilaments (Figures 6(a) and 6(b)). In contrast, the cytoplasm of leiomyoma cells has a more heterogeneous appearance due to the presence of more abundant endoplasmic reticulum, which is often swollen, and the frequent presence of vacuoles, lysosomes, and swollen mitochondria. There is a clear reduction in the thin filaments, and the associated dense bodies, in the tumor cells, when compared to myometrial smooth muscle cells. Concomitantly, a prominent increase in the endoplasmic reticulum is seen in the cytoplasm of the tumor cells, often to the extent that this organelle occupies most of the central portion of the cell, with the fine filaments relegated to the periphery of the cell (Figure 6(d)). It is also our impression that the tumor cell mitochondria, although frequently swollen, are reduced in number in comparison with the myometrial cells. For example, the large clusters of mitochondria which are sometimes seen in myometrial smooth muscle cells have not been observed in the leiomyoma cells in our samples. And it is these three changes, that is, the reduction in myofilaments, the increase in endoplasmic reticulum, and the possible reduction in mitochondria that are ultrastructural features of the phenotypic transformation from a myometrial contractile cell to a fibroid synthesizing cell.

An associated morphologic consequence of this transformation, seen with both the light and electron microscope, is that the transformed cells have less cytoplasm because of this reduction in myofilaments, and thus have a more slender, streamlined appearance. The nuclei also appear to be thinner and often have pointed ends, in contrast to the more frequently rounded ends of the myometrial cells (Figures 6(c) and 6(d)).

### 3.3. Phases 3 and 4: Excessive Synthesis of Collagen Leads to Atrophy, Injury, and Inanosis

#### 3.3.1. Atrophy

**Figure 7:**
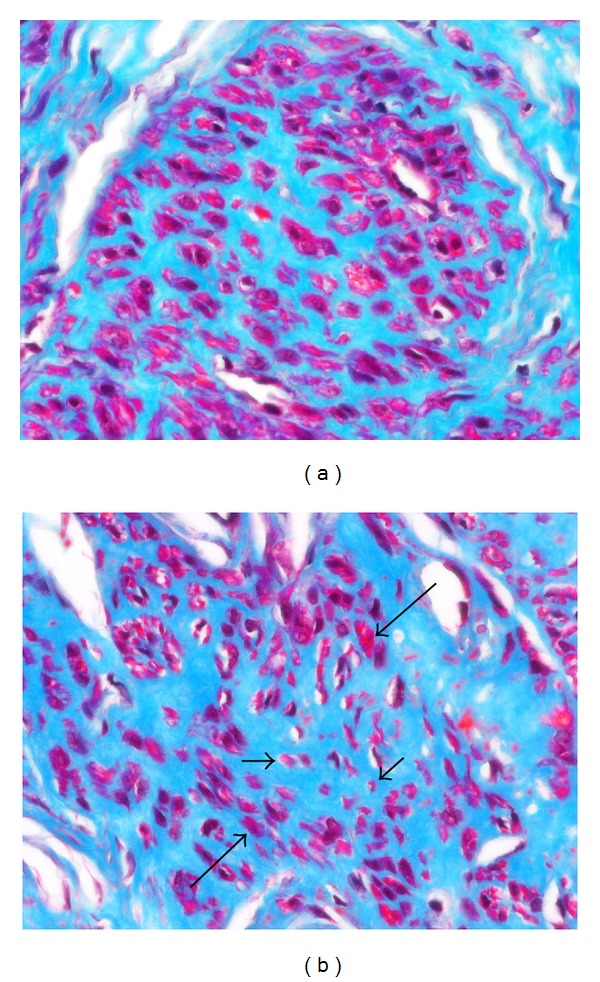
Collagen production by tumor myocytes. (a) In this Masson trichrome stained section, the interstitium of the tumor fascicle is expanded by abundant blue collagen that separates the red staining fibroid myocytes. No interspersed fibroblasts are noted in the blue collagenous stroma, indicating that the stromal matrix has been produced by the tumor myocytes themselves. (b) Many of the myocytes in the central portion of this Masson trichrome stained section are reduced in size (short arrows) in comparison with those above and below (long arrows). These appear to be atrophic cells, although the possibility of artifactual tangential sectioning cannot be excluded. Original objective magnification of (a) and (b): 40x.

**Figure 8:**
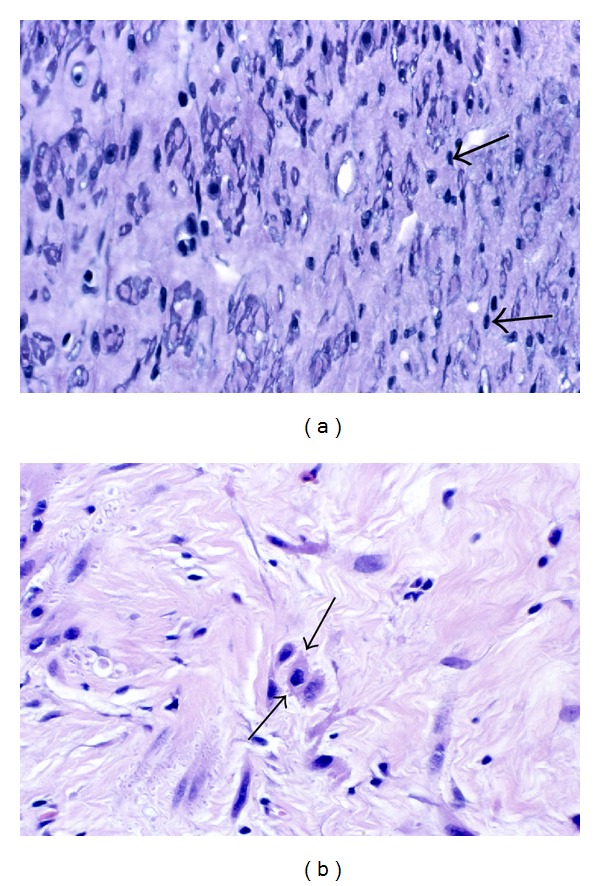
(a) Wispy atrophy of tumor myocytes. As collagen production and deposition continues, the tumor myocytes become further encased in matrix, progressively more atrophic, and often reduced to slender, wispy structures. Note that not only the cytoplasm but also the nuclei of some cells appear to be reduced in size (arrows). (b) Lateral buds versus pinnate atrophy. Sometimes the atrophic cells exhibit lateral projections (arrows), which are similar to the previously described lateral buds associated with phenotypic transformation (Figure 7), but are occurring late in the atrophic process. Whether these lateral projections are the residua of lateral buds, or whether they might represent a form of decapitation budding associated with the atrophic process (pinnate atrophy) is uncertain. Original magnification of (a) and (b): 132x.

By the examination of Masson's trichrome stained sections, it is apparent that the collagen in fibroids is produced by the myocytes themselves since there are no interspersed fibroblasts between the myocytes (Figure 7(a)). In association with the increasing collagen deposition in the later phases (Phases 3 and 4) of fibroid development, it is also evident that myocyte atrophy is occurring (Figure 7(b)). This is sometimes evident by comparing one fascicle of myocytes with another. The atrophic process involves a reduction in size of both the cytoplasm and the nucleus of myocytes. In the late stages of atrophy, the cells are often reduced to slender, elongate, wispy structures (Figure 8(a)). Some of the atrophic myocytes will be noted to exhibit changes in shape, such as the presence of lateral pseudopodal projections or buds (pinnate atrophy) (Figure 8(b)). Whether the latter represent a form of decapitation membrane budding (similar to apocrine secretion) associated with the atrophic process, or alternatively the remnants or the shedding of intercellular nexus attachments (gap junctions) as the cells transform from myocytes to fibroblast-like cells is not clear.

**Figure 9:**
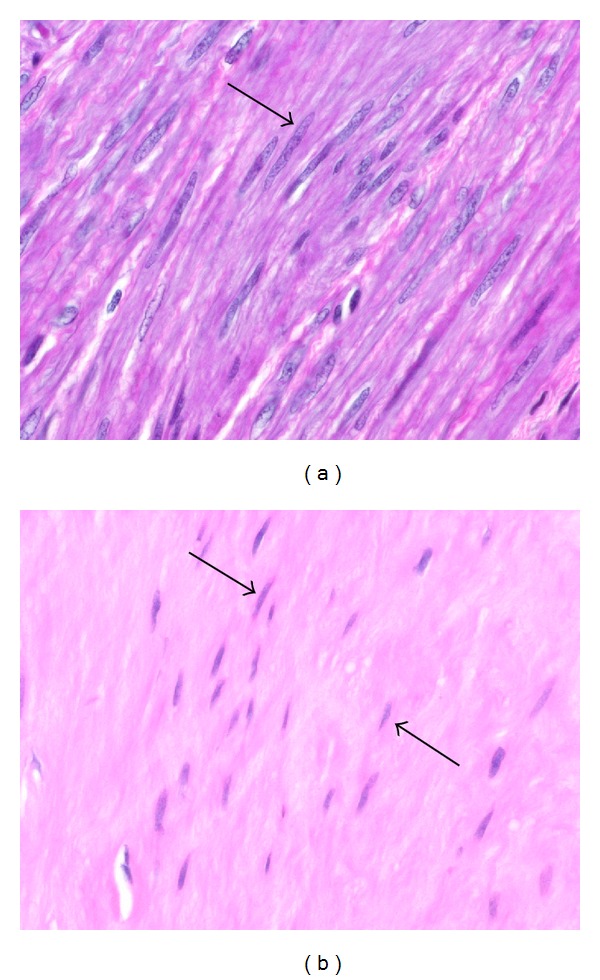
Cytoplasmic and nuclear atrophy. The eosinophilic cytoplasm of the fibroid myocytes in (b) is indistinct but appears diminished and less abundant than that of the myometrial myocytes in (a), and there is an obvious increase in the stroma that is separating the fibroid cells of this Phase 4 tumor. In addition, the nuclei of the myometrial cells in (a) are uniformly long and straight (arrow), while the fibroid nuclei in (b) exhibit variation in length and contour (arrows) and often appear to be shortened, suggestive of early nuclear shrinkage. Original objective magnification of both (a) and (b): 40x.

**Figure 10:**
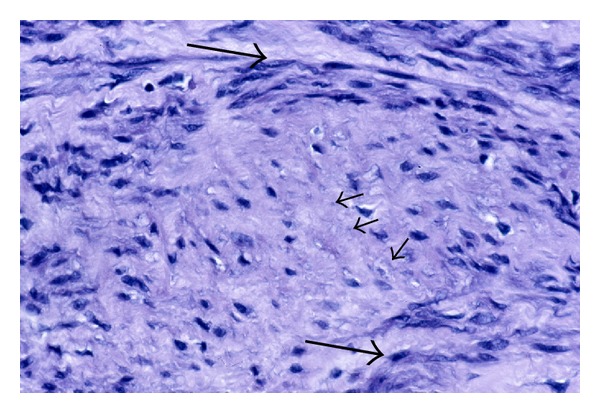
Nuclear atrophy. In this image from a late phase fibroid, the myocytes at the top and lower right (long arrows) retain some visible cytoplasm and nuclear length, while the cells in the center of the fibrotic matrix show little remaining cytoplasm and nuclei that are small, variably shaped, and appear to be atrophic. In addition, near the most central portion of this image there are a few pale staining, barely visible nuclei of cells that are assumed to be nonviable (short arrows). Original magnification: 132x.

Although the nuclei of fibroid myocytes seem to retain their size and shape during much of the atrophic process, the nuclei often appear to shrink in size during the late stages of atrophy. To make this evaluation, it is necessary to examine cells cut in the longitudinal axis, but even with this in mind it is hard to exclude the possibility of partial tangential sectioning. However, if we compare side-by-side images, taken at the same magnification, of nonatrophic myometrial myocytes (Figure 9(a)) with fibroid myocytes from an atrophic area in a Phase 4 fibroid (Figure 9(b)), there does appear to be reduction in the length and width of many of the fibroid myocyte nuclei. Using an ocular micrometer, most of the measurable nuclei in the myometrial image (Figure 9(a)) ranged from 22.5 to 27.5 *μ*m in length, while the nuclear lengths of the fibroid nuclei in this image (Figure 9(b)) measured from 10.0 *μ*m to 20.0 *μ*m, with most of the latter measuring between 12.5 and 15.0 *μ*m. In later stages of atrophy, smaller nuclei may be noted (Figure 10). The apparent nuclear atrophy would imply that some loss of nucleoplasm is consistent with viability, although there is obviously a limit to nuclear atrophy, just as there is a limit to the loss of the metabolic and synthetic organelles of the cytoplasm.

#### 3.3.2. Injury (Degenerative Change)

**Figure 11:**
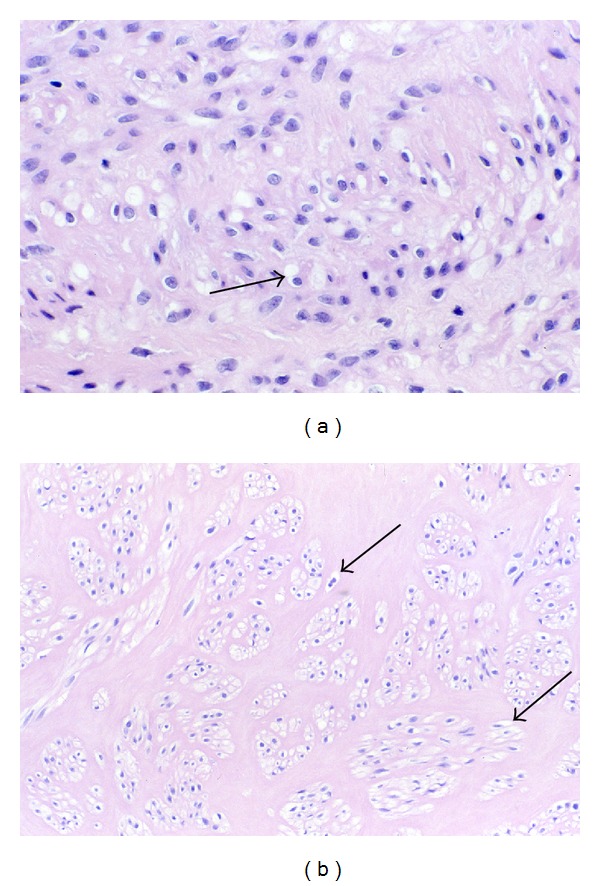
Vacuolization of myocytes. In Phase 3 and 4 fibroids, cytoplasmic vacuolization (arrows) is common and may reflect the loss of myofilaments and other changes related to injury or involution. As noted in both (a) and (b), the myocytes are often surrounded by an amorphous (hyaline) matrix. Original magnification of (a): 132x. Original magnification of (b): 66x.

In addition to shrinking in size, the myocytes often exhibit cytoplasmic vacuolization, particularly in the Phase 3 and 4 tumors (Figure 11). The vacuolization may take the form of diffuse cytoplasmic pallor surrounding a central or eccentric nucleus, or there may be one large cytoplasmic vacuole that appears to displace the nucleus to one side of the cell. In some tumors, several large foci or clusters of multiple vacuolated cells may be seen (Figure 11(b)). In view of the association of this change with the later phase tumors that are also exhibiting atrophic features, it seems likely that the vacuolization is related to the involutional changes that are occurring in these tumors. That is, the vacuoles could represent either swollen endoplasmic reticulum due to injury or possibly active synthesis [11], or phagolysosomes or autophagic vacuoles as the atrophic cells dispose of nonessential organelles and myofilaments [12].

**Figure 12:**
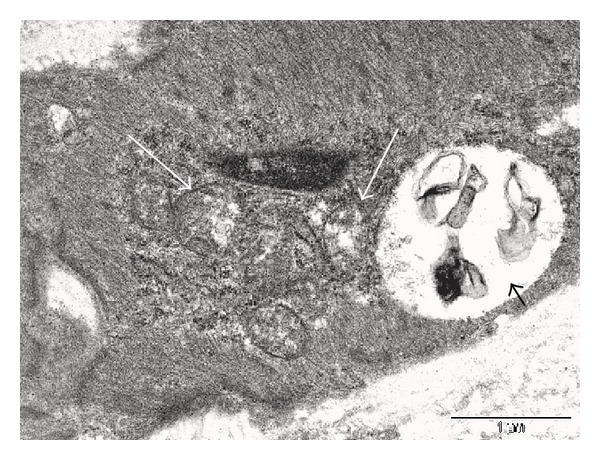
Injury and autophagy. A cluster of degenerating mitochondria (long arrows) near the center of the image are swollen and show fragmentation and loss of cristae. The myofilaments of the surrounding cytoplasm also appear granular and degenerate. To the right of the mitochondria, there is vacuole, probably autophagic, containing membranous debris (short arrow). The image suggests that the degenerate mitochondria and myofilaments are being drawn into an autophagic vacuole. Original magnification: 20,500x.

**Figure 13:**
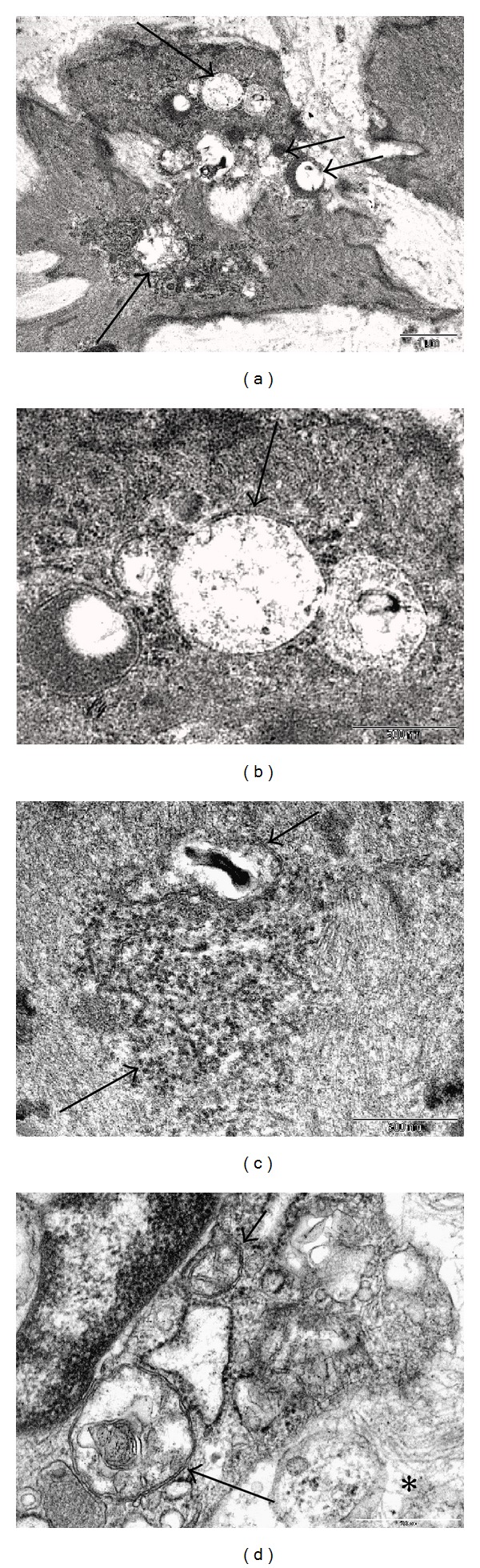
Lysosomes and Autophagy. The myocyte in (a) contains several vacuoles (long arrow at top and left lower) and lysosomes (shorter, horizontal arrows in middle). Higher magnification of the 4 structures at the top of the cell in (b) shows partial fusion of vacuoles to form a double membraned autophagosome in the middle (arrow). Granular debris in the vacuolar lumens may represent degenerate myofilament particles. The image in (c) shows degenerate myofilaments in the center (long arrow) and an autophagic vacuole (short arrow) which appears to be engulfing the degenerate filaments. The myofilaments on either side of this central area appear to be intact. The cell in (d) exhibits more advanced changes with loosely expanded cytoplasm separating the myofilaments on the right (asterisk), degenerating and swollen mitochondria (short arrow), and an autophagosome with membranous and particulate debris (long arrow). Original magnification of (a): 11,500x. Original magnification of (b), (c), and (d): 43,000x.

Examination of fibroids by electron microscopy offers evidence that both injury and autophagocytosis are occurring. In addition to the swelling of endoplasmic reticulum and mitochondria in leiomyoma cells, the mitochondrial cristae are often decreased and fragmented (Figure 12). Lysosomes and autophagic vacuoles also appear to be increased (Figures 13(a) and 13(b)), sometimes containing myelin figures and sometimes lying adjacent to mitochondria or degenerating myofilaments, suggesting that these structures are being engulfed by the autophagic vacuoles. These changes support the concept of atrophy with autophagic disposition of myofilaments and nonessential organelles, as well as injury, in later stage fibroids (Figures 13(c) and 13(d)).

#### 3.3.3. Vascular Changes in Fibroids

**Figure 14:**
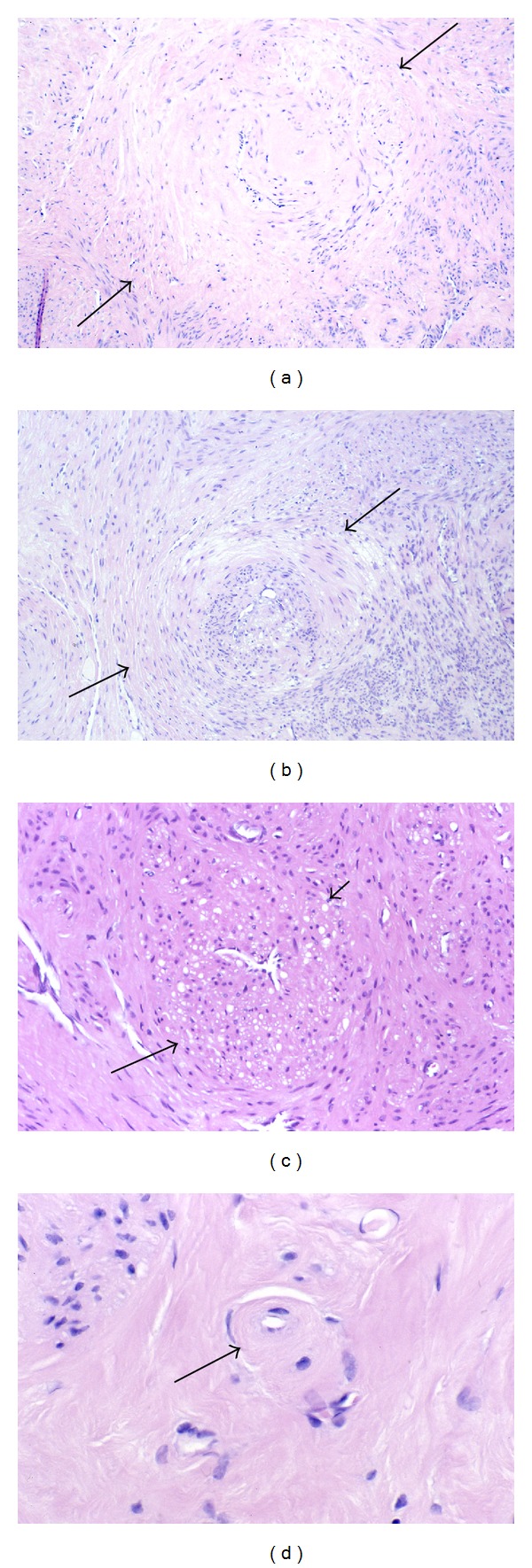
Vascular changes within fibroids mirror those of the fibroid tumor cells. Atrophy of the tumor myocytes occurs as a result of both vascular and interstitial changes. The vascular smooth muscle cells within fibroid tumors often exhibit changes similar to those occurring in the tumor myocytes, including hypertrophy and hyperplasia of the medial myocytes (a), medial hyperplasia and intimal fibrosis (b), vacuolization (c), and hyalinization (d). Arrows mark the outer perimeters of the thickened vessels. Note that each of these vessels also shows marked stenosis of the lumen, which contributes to the ischemic, atrophic process. Original magnification of (a): 33x, (b): 33x, (c): 66x, and (d): 132x.

The myocytes within the blood vessels of fibroids frequently exhibit changes that mirror those of the tumor myocytes. Medial hypertrophy of vessels due to smooth muscle cell proliferation is commonly seen (Figure 14(a)). Similarly, in tumors displaying fibrosis, there is often a corresponding fibrosis of the blood vessel walls, either medial or intimal (Figure 14(b)). Other changes noted in both the vessels and the tumor itself include vacuolization (Figure 14(c)), hyalinization (Figure 14(d)), mucinosis or myxoid change, and myocyte atypia or symplastic change, characterized by nuclear enlargement and hyperchromasia. The consequence of these vascular changes, such as the smooth muscle hyperplasia, fibrosis, or hyalinization, is progressive stenosis of blood vessel lumens, which contributes to the ischemic environment that results in eventual atrophy of tumor myocytes. In addition to the reduced blood flow from stenosis of the lumens, hyalinized arterioles are probably also unable to dilate and thus unable to increase the blood flow in response to the low oxygen tension.

#### 3.3.4. Inanosis

**Figure 15:**
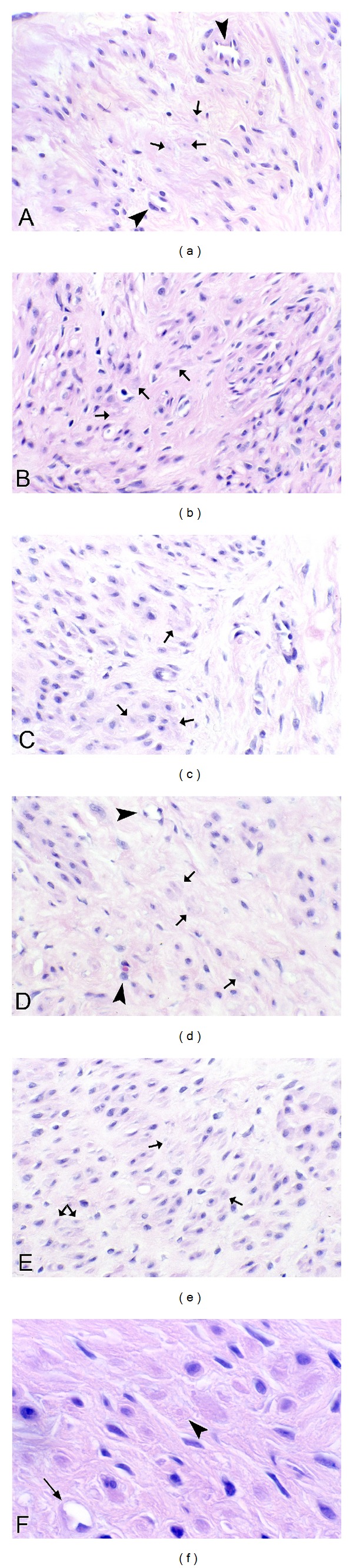
Inanosis of tumor myocytes. Interstitial and vascular ischemia lead to myocyte atrophy and eventual cell death, a process that we refer to as inanosis. In this panel, each image shows cytoplasmic and nuclear atrophy of tumor myocytes, as well as scattered, shrunken cells with marked nuclear pallor that are probably nonviable (arrows in (a)–(e)). In addition to those marked by arrows, there are other similar pale, presumably nonviable cells scattered among the viable cells with blue nuclei, resulting in a mottled pattern. Note the absence of inflammatory reaction. (a) The pale, inanotic cells are widely separated from the capillary at the top and the capillary at the bottom (arrowheads). (b) The pale, presumably dead cells are scattered among atrophic, but viable, cells with dark blue nuclei. (c) The pale, inanotic cells are shrunken but usually maintain elongate shapes. (d) Note that the pale, inanotic cells in the center are the most distant cells from the capillary with the open lumen at the top and the apparent capillary with a red cell in the lumen at the bottom (arrowheads). (e) Pale cells are noted here and there among the atrophic, viable cells in a field without any obvious capillaries. No inflammation is present. Original magnification of photos ((a)–(e)): 132x. (f) Oil immersion image of inanotic cells (myocyte tombstones) with pale nuclei and cytoplasm, in a fibrotic, atrophic field of a Phase 3 tumor. The cell in the center of the field (arrowhead) is located 76 *μ*m from the capillary in the left lower corner (arrow). Original magnification of (f): 330x.

Within fibroid tumors that have accumulated extracellular collagenous matrix—that is, Phase 3 and 4 tumors—there are almost invariably found a few small, pale shrunken myocytes (Figure 15). Because of the small size and pale staining nucleus and cytoplasm of these cells, they are inconspicuous. They are most commonly found in small clusters of two to four cells entrapped within the areas of most abundant collagenous matrix, which is usually of the amorphous or hyaline type. These cells are only a fraction (1/3 to 1/5) of the size of normal myocytes. Their nuclei are tiny, rounded to ovoid, pale staining, and never fragmented. Nucleoli are not seen. Their cytoplasm retains an ovoid to fusiform shape, is very pale to lightly eosinophilic, and is in contact with the extracellular matrix. Since each of these cells is surrounded and insulated by the interstitial matrix, they may lie in proximity to each other, but they are usually not contiguous.

Within a given field, these shrunken cells are generally similar in size and tinctorial qualities, although size and tinctorial preservation of these cells will vary from one focus to another. That is, some cells may be markedly reduced in size but retain slight basophilia of the nucleus, whereas the shrunken cells in other areas will exhibit nuclei which are even more diminutive and so pale as to be barely visible. When measured with an ocular micrometer, the smallest nuclei will measure from 1 to 2 *μ*m in diameter. The shrunken cells will usually lie at considerable distance from the nearest visible capillaries, separated by more than 30 *μ*m and sometimes by as much as 50 *μ*m from the closest vessels (Figure 15(a)). Thus, it appears in many tumors that as the quantity of collagenous tissue increases, the microvessel density correspondingly decreases. It is also evident that there is never any observed phagocytosis of these cells nor any adjacent inflammatory response.

Although the argument could be made that these small, pale cells represent tangential cuts of larger cells, it is clear that atrophy of myocytes is occurring since other myocytes in these hyalinized areas are also reduced in size—but to a lesser degree and with retention of normal nuclear basophilia. In addition, it would seem unlikely to have so many small, pale nuclei in one field as a result of tangential sectioning.

The mildly to moderately atrophic myocytes with retention of normal nuclear staining are probably viable. The viability of severely shrunken cells with retention of some nuclear staining is questionable. On the other hand, those cells exhibiting the most extreme degrees of nuclear and cytoplasmic atrophy, with such loss of nuclear staining that the nuclei are barely visible, are assumed to be nonviable. These latter cells are regarded as myocyte tombstones, and they are the histologic hallmark and end stage of the atrophic process that we have designated as inanosis.

The term inanosis denotes a condition of cellular inanition resulting from gradual nutritional deprivation. This catabolic process of severe atrophy, eventuating in cell death by inanosis, is the consequence of the elaboration of extracellular matrix within the tumor as well as within the vessel walls of the tumor. Blood flow is diminished by progressive stenosis of vascular lumens resulting from the intratumoral vascular smooth muscle proliferation and fibrosis that mirrors the pathology of the fibroid tumor itself. Of equal if not greater import, however, is the interstitial fibrosis occurring within and between the fascicles of tumor myocytes themselves. The consequence of this process is the progressive separation of myocytes from capillaries, thus increasing the diffusion distance for vital nutrients to reach the myocytes from the capillaries, eventually resulting in atrophy and ultimately in cell death. This condition of interstitial ischemia is probably exacerbated by the dense collagenous character of the fibroid extracellular matrix, which most likely contains less ground substance for the diffusion of oxygen and nutrients.

#### 3.3.5. Postinanotic Changes (Reclamation)

**Figure 16:**
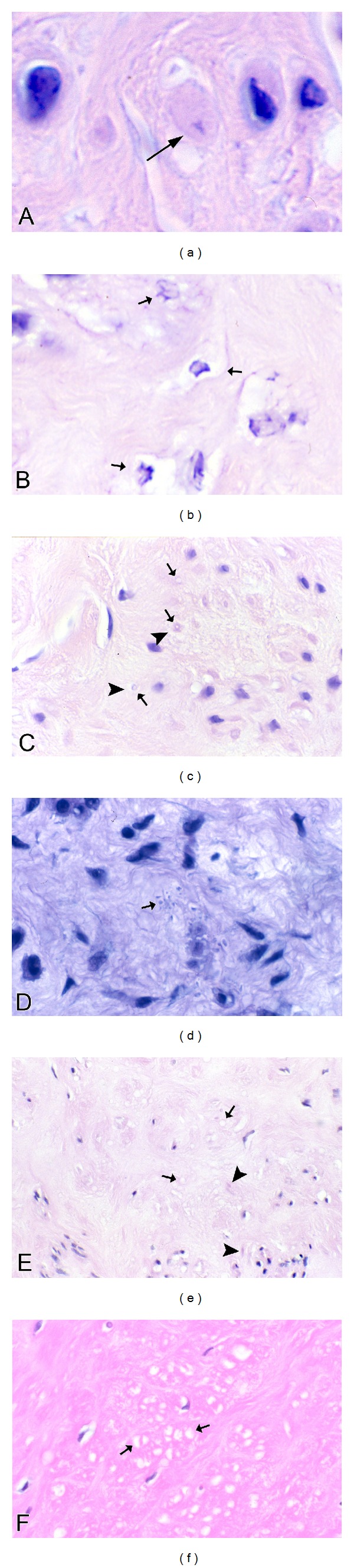
Reclamation. Cytologic and histologic changes are sometimes noted in involuting fibroids that are suggestive of cell resorption, a process that we refer to as reclamation. Angulated nuclear shapes, such as the triangular shaped nucleus in (a) (arrow) and the burr-shaped nuclei (acanthanuclear alteration) in (b) (arrows), are aberrant shapes that would not be expected in viable cells and may be related to loss of nuclear membrane integrity. Structures that appear to be hollow or empty nuclei, with only a shell of surrounding cytoplasm, are noted in (c) (arrows), and these structures often lie within clear spaces in the stroma that may represent resorption pits (arrowheads). Small fragments of probable cytoplasmic material are sometimes noted within clearings in the stroma, as seen in (d) (arrow); whether these are discarded lateral buds or decapitated particles of cell cytoplasm is uncertain. Finally, clear, circular spaces are sometimes noted within hyalinized areas of Phase 4 tumors, as seen in (e) and (f) (arrows) that are believed to be the end stage of the reclamation process. In (e), inanotic dead cells are also noted (arrowheads). In (f), the clear spaces are numerous, resulting in a spongiform appearance. Original magnification of (a): 330x, (b): 330x, (c): 330x, (d): 198x, (e): 132x, and (f): 40x.

A variety of changes are noted in both the nonviable cells and the surrounding stroma of involuting fibroids that are suggestive of resorption of the nonviable cells, a process that we refer to as reclamation. Although most inanotic nuclei are small, rounded structures, some exhibit sharply angulated shapes, one of the more frequent of these being the triangulated nucleus (Figure 16(a)). Such a nuclear shape would be unusual for a normal, viable cell. In addition, some inanotic cells are seen with nuclei exhibiting two or more pointed projections resembling burrs (Figure 16(b)). It is possible that these abnormal nuclear shapes could be artifacts of processing or sectioning, although that seems less likely for the burr-shaped nuclei. We refer to the burr-shaped nuclei as showing acanthanuclear alteration by way of analogy to the acanthocytic red blood cells seen in certain pathologic states such as severe hepatocellular injury and the genetic disorder abetalipoproteinemia [13]. Since the abnormal red blood cell shapes in these latter disorders are related to aberrations in the relative amounts of cholesterol and specific phospholipids in the lipid bilayers of the plasma membrane, we have considered the possibility that a similar phenomenon could be responsible for the irregular shapes occurring in some inanotic nuclei. Such distortions in the nuclear membranes of presumed nonviable inanotic cells could be related to a loss of nuclear membrane integrity, secondary to extraction of membrane components for recycling purposes.

More advanced degrees of reclamation are suggested by fields in which inanotic cells exhibit hollow or empty nuclei, with only shells of cytoplasm remaining (Figure 16(c)). Fragments of apparent cytoplasmic debris, resembling the previously described lateral buds, but possibly representing decapitated segments of cytoplasm associated with atrophy, are occasionally noted in the stroma (Figure 16(d)). Both the hollow nuclei and the cytoplasmic fragments may lie within clear spaces in the stroma that are believed to be resorption pits. Finally, in the hyalinized areas of some Phase 4 fibroids, the tissue exhibits occasional foci with a spongiform or motheaten appearance due to the presence of multiple empty spaces. These spaces are believed to be the sites of complete resorption of inanotic cells and cell debris and thus are thought to represent the end stage of reclamation (Figures 16(e) and 16(f)).

**Figure 17:**
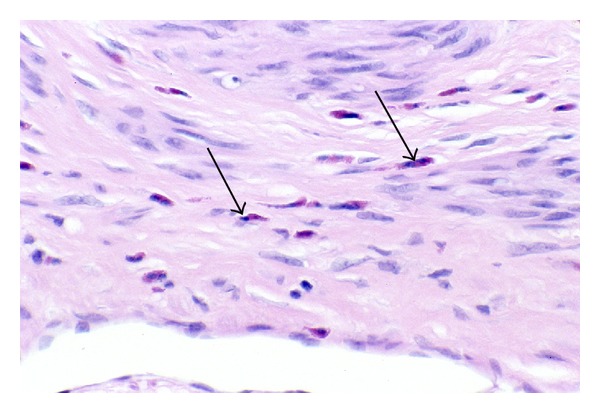
Leukocytes traversing fibrotic stroma of a fibroid tumor. Multiple ameboid eosinophils (arrows) with filopodial extensions are traversing the fibrotic stroma of this fibroid, seemingly unimpeded. Foci such as this indicate that the fibrotic stroma of fibroids can be penetrated by granulocytes and weakens the argument that the lack of inflammatory response to the dead and fragmented inanotic cells is due to the physical resistance of the dense fibrotic stroma in fibroids. Original magnification: 132x.

An interesting feature of reclamation is the lack of an inflammatory reaction or recruitment of macrophages. We have considered the possibility that it might be difficult for inflammatory cells to pass through the dense collagen of these fibrotic areas; however, the finding of leukocytes with filopodial extensions traversing through the stroma of some fibroid tumors (Figure 17) indicates that at least some fibroids are accessible to the influx of inflammatory cells. Since there is no accompanying inflammatory reaction or evidence of phagocytosis, we feel that this is a process distinct from that normally associated with reaction to necrotic or apoptotic cells. Thus we refer to this process of non-phagocytic, presumably enzymatic, resorption and recycling of cellular components as reclamation since the body is reclaiming the molecular contents for use by other cells.

### 3.4. Transmission Electron Microscopy (TEM) of Fibrotic Areas

**Figure 18:**
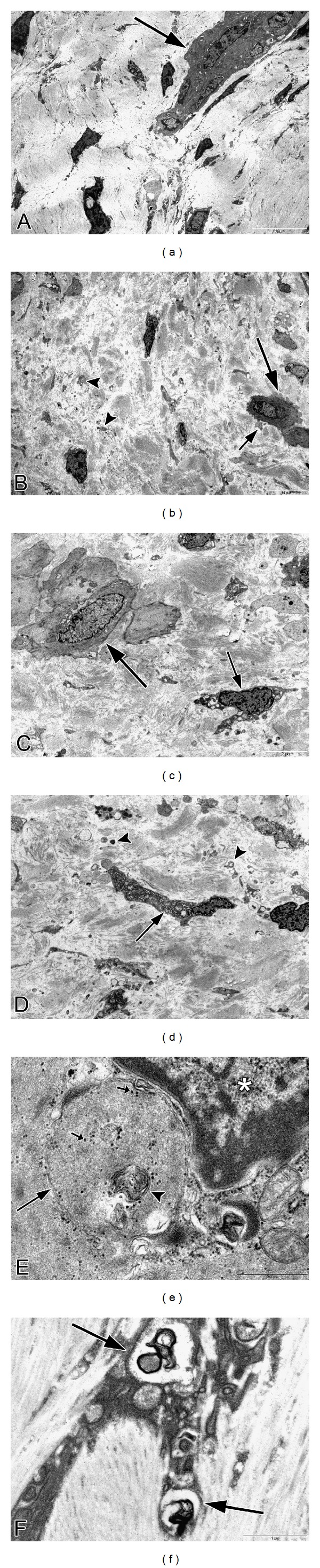
Ultrastructure of inanosis and reclamation. Electron microscopic examination of fibrotic areas in fibroids provides even more dramatic evidence of shrunken myocytes, widely spaced within the fibrotic stroma. In (a), there is a cluster of cells which have retained some cytoplasm (arrow) and provide a frame of reference for the small, shrunken cells. In (b), a cell in the lower right (long arrow) exhibits cytoplasmic budding, with one or two of these knob-like fragments apparently pinching off from the cell membrane (small arrow); we refer to this as decapitation membrane budding. Note that the loose, watery stroma contains numerous particles of cellular debris (arrowheads), without any evidence of inflammatory cell infiltrate or phagocytosis. In (c), the shrunken cell in the lower right (short arrow) contrasts with the less atrophic cell in the upper left (long arrow). The cytoplasm of the shrunken cell contains vacuoles, lysosomes, and degenerating organelles with few remaining myofilaments. The slender, atrophic cell in the center of the fibrotic field in (d) has retained its shape and endoplasmic reticulum (arrow), but has lost most of its myofilaments. The surrounding matrix contains abundant cellular debris (arrowheads), imparting a junkyard appearance. In (e), there is an autophagosome (long arrow) containing ribosomes (short arrows) and membranous debris which could be of endoplasmic reticulum origin (arrowhead). The remainder of the contents of the autophagosome resembles the adjacent cytosol and probably consists of degenerated myofilaments. The nucleus (asterisk) is in the upper right of the image. The cell in (f) exhibits two double membraned autophagosomes (arrows) containing electron-dense membranous debris. Original magnification of (a): 1700x, (b): 1250x, (c): 2550x, (d): 2550x, (e): 43,000x, and (f): 20,500x.

Ultrastructural analysis of fibrotic areas reveals much more than can be appreciated by light microscopy. Thin, wispy atrophic cells, degenerating cells, and remnants of dead cells can be found entrapped in the dense collagenous matrix (Figures 18(a) and 18(b)). Many of these cells or cell fragments are too small to be visualized with the light microscope. Occasional cells show membrane budding with some of the buds loosely attached (Figure 18(b)), suggesting that this may be a form of decapitation membrane budding associated with atrophic downsizing. Some degenerating cells contain swollen endoplasmic reticulum and degenerating mitochondria that largely fill the cytoplasm. Fine myofilaments are prominently reduced (Figures 18(c) and 18(d)). Lysosomes often appear increased. Irregular vacuolar structures containing cytoplasmic debris and probably representing phagolysosomes are sometimes seen within the cytoplasm. Double membraned autophagic vacuoles are occasionally encountered (Figures 18(e) and 18(f)), frequently containing membranous debris and sometimes containing ribosomes or the stacked membranes of the Golgi apparatus. These changes are consistent with the atrophic and vacuolar degenerative changes noted with the light microscope and presumably reflect both atrophic downsizing as well as ongoing degeneration related to ischemia and nutritional deprivation.

**Figure 19:**
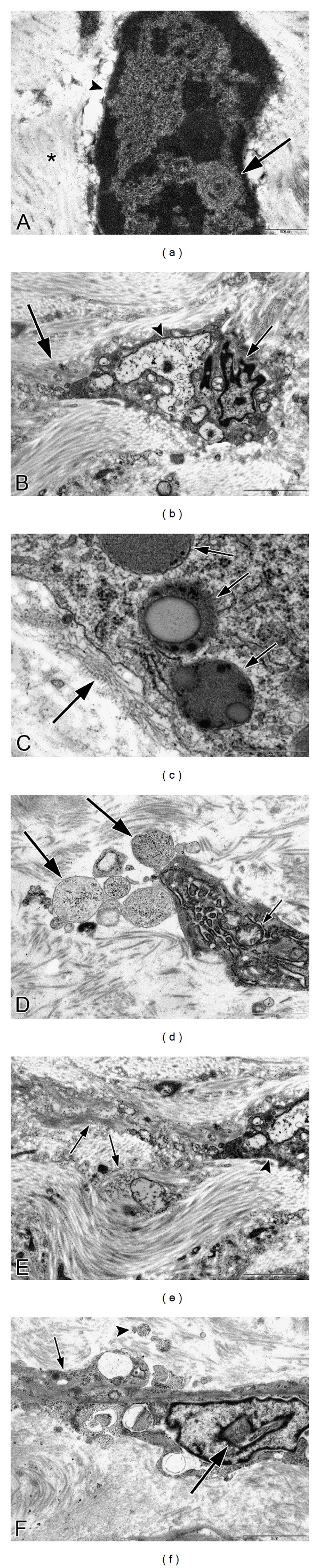
Ultrastructure of inanosis and reclamation. The nucleus in (a) is lying free within the stroma and shows a swollen, discontinuous nuclear envelope (arrowhead) that is being resorbed. The nucleus itself appears relatively intact except for the whorl in the lower right (arrow). Note the loosely expanded, watery stroma (asterisk) surrounding the nucleus. The nucleus of the shrunken cell in (b) displays a rippled or serrated border (short arrow) resembling the acanthanuclear alteration sometimes seen with the light microscope. The large vacuolar structure in the cytoplasm (arrowhead) could be dilated endoplasmic reticulum or possibly a phagolysosome and may correspond to the cytoplasmic vacuoles seen with the light microscope. The cytoplasmic tail at the left (long arrow) is degenerated and could be a precursor to autoamputation. In (c), myofilaments (long arrow) appear to lie outside of the cell membrane within a loosely expanded, watery stroma, suggesting that they have either leaked out or have been extruded. Three phagolysosomes (short arrows) are present in the cytoplasm of the cell. The cell in (d) shows budding of cytoplasmic debris (long arrows) from the elongate end of the cell. Some of the rounded particles could be autophagolysosomes with free ribosomes and degenerated cytoplasm. The cytoplasm of the cell consists almost entirely of dilated ER and swollen mitochondria (short arrow), with few remaining filaments. The tail of the cell in (e) exhibits complete degeneration of cytoplasm and organelles (arrows), with apparent loss of the cell membrane, while the remaining portion of the cell on the right (arrowhead) appears to be viable; the appearance suggests that cells might be capable of segmental autoamputation. The cell in (f) illustrates both the end stage of inanosis and the resorptive process of reclamation. The cytoplasmic contents are degenerated, granular, and vacuolated (short arrow), and the cell membrane has been breached in some areas, resulting in extrusion of degenerated cytoplasm into the adjacent watery stroma with disrupted collagenous matrix (arrowhead). The nuclear chromatin is condensed, and there is a degenerative structure in the middle (long arrow). Note that in each photo ((a)–(f)), the inanotic cells or cell particles are surrounded by loosely expanded, watery stroma, which is believed to correspond to the clear spaces sometimes seen around inanotic cells with light microscopy and thought to represent resorption pits associated with the reclamation process. Original magnification of (a): 26,500x, (b): 9,900x, (c): 43,000x, (d): 9,900x, (e): 9,900x, and (f): 9,900x.

Frequently, nuclei are not seen in these degenerating cells, although this could be related to tangential sectioning. Atrophic, but otherwise intact appearing, nuclei may be seen in markedly atrophic cells with greatly reduced cytoplasm that is largely degenerated, indicating that the nucleus is capable of downsizing and also that it is probably the last organelle to degenerate. Isolated, or naked, nuclei may be seen without surrounding cytoplasm, indicating that both the nuclear and cytoplasmic fragments may be remnants of dead cells (Figure 19(a)). Occasional degenerating cells with small nuclei exhibiting irregular, undulating or serrated borders are noted; this is an appearance similar to that of the acanthanuclear alteration noted with the light microscope and described above (Figure 19(b)).

Another feature often noted with TEM of fibrotic areas in fibroids is that fine filaments and cytoplasmic organelles such as mitochondria and ribosomes may be seen outside of any enclosing cytoplasmic membrane, lying free within the surrounding collagen fibers (Figure 19(c)). Although we have questioned whether this appearance is due to tangential sectioning, the myofilaments and other structures are sometimes seen adjacent to collagen fibers, thus offering support for an extracellular location, and they are often associated with adjacent cells that appear viable (Figure 19(d)). It seems more likely that this release of cellular structures is indicative of leaky cell membranes (degradative porosity) associated with dead or dying cells during the postinanotic state of reclamation, but the possibility cannot be excluded that this could be a mechanism of inanotic expulsion that might occur in the atrophic, inanotic phase. Nevertheless it is notable that despite the presence of cell organelles and cytoplasmic particles lying within the extracellular matrix, no inflammatory cells are present, and no macrophages with apoptotic bodies are noted. This suggests that cytokines have not been released during the slow, progressive process of inanosis, which now may be complete and giving way to an orderly disposal process of reclamation.

In addition, occasional cells exhibit entire segments of cytoplasm in which both the organelles and the cytoplasm itself are degenerated and are lacking a well-defined surrounding plasma membrane. This latter appearance, as well as the presence of islands of degenerated cytoplasmic fragments in the collagenous stroma, suggests that atrophic, degenerating cells may be capable of autoamputation of segments of cytoplasm (Figure 19(e)). Thus, atrophy of cells may involve multiple processes, including lysosomal and autophagosomal digestion of organelles and other constituents, decapitation membrane budding, and possibly expulsion of cell organelles and filaments and/or segmental autoamputation.

Reclamation, or the non-phagocytic resorption of dead cells and cell fragments, can be readily appreciated in the fibrotic areas of leiomyomas. In fact, in almost any fibrotic field, there will be fragments of cells or cytoplasmic particles dispersed within the stroma that are indicative of cell breakdown or expulsion, in the absence of inflammatory reaction (Figures 18 and 19). In addition to the dispersed fragments of cell cytoplasm and organelles lying free in the collagenous matrix, naked nuclei are sometimes seen (Figure 19(a)), with the latter often showing swollen or incomplete nuclear membranes and degenerated, clumped chromatin. Disintegrating cells with absence of plasma membrane in focal areas will be seen (Figure 19(f)). Characteristically, both the naked nuclei and the disintegrating cells are surrounded by clear zones with reduced collagenous matrix (Figures 19(a)–19(f)). These clear zones are believed to be resorption sites, similar to the resorption pits produced by osteoclasts or the digestion chambers of degenerating nerve fibers, in which organic material is digested by proteases. Both matrix metalloproteinases and lysosomal enzymes released from the disintegrating cells could be sources of this toxic milieu, which will ultimately result in the degradation of cellular material to the molecular level, so that it can be absorbed into the circulation and recycled. Thus, the images speak for themselves: degradation of cells and cellular contents is clearly occurring in these fibrotic areas without the assistance of phagocytic cells.

**Figure 20:**
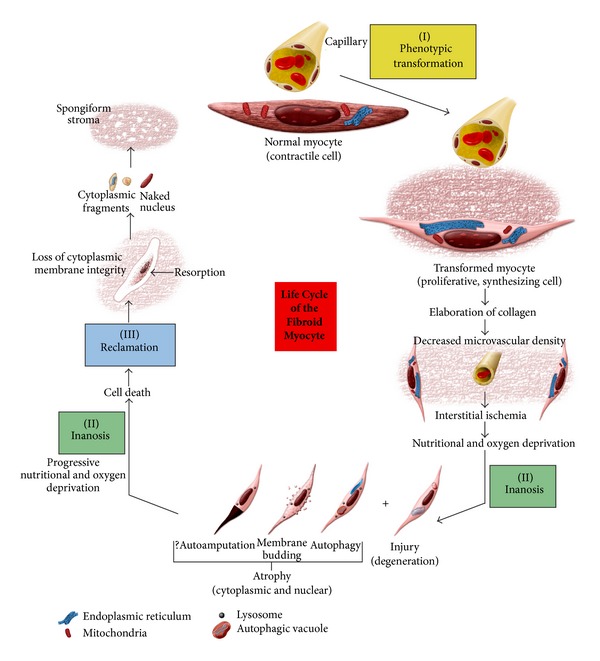
Life cycle of the fibroid myocyte. In this graphic representation, we have attempted to summarize our impression of the fibroid myocyte life cycle, from (I) phenotypic transformation to a proliferating, synthesizing cell, to (II) inanotic injury, atrophy, and ultimately cell death resulting from interstitial and vascular ischemia, to (III) reclamation in which dead cells and cell contents are resorbed by enzymatic degradation without the involvement of phagocytic cells. Illustration by David Sabio, Experimental Pathology Laboratories.

In summary, based upon our observations and our interpretation of these findings, as reported above, we have attempted to illustrate the concepts of phenotypic transformation, interstitial ischemia, inanosis, and reclamation in a graphic representation (Figure 20).

## 4. Discussion

Our observations suggest that many and probably most fibroids pursue a self-limited life cycle, which may be arbitrarily divided into 4 Phases (Table 1). Phase 1, in which no, or insignificant, collagen matrix is present, is characterized predominantly by proliferation of myocytes. Similar to the experimentally-induced injury response of vascular smooth muscle, the proliferation of uterine myocytes may represent an injury response to the hypoxia of vasoconstriction occurring during menstruation, especially in women with the myometrial hypercontractility associated with dysmenorrhea. Under the continual stimulus of estrogen and progesterone, the smooth muscle cells continue to proliferate, eventually becoming biclonal or monoclonal as individual cell types with growth advantages emerge. Eventually and perhaps even at the onset in some tumors, the proliferating myocytes begin to elaborate collagen (Phase 2 = <10% collagen), completing the injury response in which smooth muscle cells are transformed from a contractile phenotype to a proliferative/synthetic phenotype. In Phase 3, proliferation continues, but the balance now tilts progressively in favor of collagen synthesis (10–50% collagen), and the extracellular matrix begins to accumulate and even predominate. Not only are the myocytes now further removed from the nearest capillaries because of the excess matrix, but also the angiogenesis seems to lag behind the growth in size of the tumor. Perhaps the production of fibrogenic growth factors, such as FGF [14] and TGF-*β*, exceeds the production of angiogenic growth factors such as VEGF, because the microvessel density appears to decline progressively as the tumors grow and age. Since all cells must lie within a reasonable distance of capillaries to receive oxygen and nutrients, eventually a state of interstitial ischemia will supervene. The histologic consequence of this interstitial ischemia is cellular atrophy, which is characteristic of late Phase 3 tumors and is one of the defining features of Phase 4 tumors.

During the final phase (Phase 4) in the life of a fibroid, the excessive production of extracellular matrix reaches a maximum (>50% collagen), angiogenesis is reduced or at least fails to keep pace with the growth of the tumor, myocyte proliferation is greatly diminished or absent, and cellular atrophy occurs. This is the phase of involution, characterized by large areas of hyaline matrix with scattered islands of atrophic myocytes. Necrosis due to infarction is often seen in Phase 4, but infarction may occur in any phase. Likewise, apoptotic cells are occasionally noted. However, a third form of cell death, inanosis, the ultimate endpoint of atrophy and the consequence of nutritional deprivation, is a characteristic and defining feature of this final phase.

In some respects, the process of inanosis occurring within fibroids can be considered ischemic in nature and in this respect likened to both necrosis and apoptosis which may occur as a result of ischemia. A fundamental difference between these processes, however, is the rapidity with which they occur. Necrosis and apoptosis are both relatively rapid consequences of severe to moderately severe ischemic injury, while the histologic features of inanosis suggest that this is a very slow atrophic process which probably progresses over days to months until cellular proteins have been depleted to the point that vital cellular functions can no longer be maintained. In fact, zones of necrosis are not infrequently found in fibroids, presumably resulting from more sudden and profound ischemia, and rare cells which appear apoptotic are noted among the myocytes of Phase 3 and 4 tumors. From a quantitative standpoint, however, the atrophic cells of inanosis are more commonly found in late phase fibroids than are apoptotic cells, and they occur more consistently than do zones of necrosis.

In addition to pathogenetic differences, the morphologic features of inanosis, particularly the nuclear changes, are distinct from those of apoptosis. Apoptosis is a well-described and morphologically defined pattern of cell death. Other than cell shrinkage, there is very little similarity between apoptosis and inanosis by light microscopy. When the apoptotic cell shrinks, it also tends to retract from the surrounding tissue, rounds up and condenses the cytoplasm, whereas the atrophic cells of inanosis shrink to a more marked degree but usually maintain their shape and their connection with the surrounding stroma. While the apoptotic cell often exhibits hypereosinophilic cytoplasm, inanotic cells typically show cytoplasmic pallor, probably because of progressive depletion of cytoplasmic protein and organelles. Moreover, the nuclear changes are particularly different in that apoptotic cells characteristically condense their chromatin in peripheral aggregates against the nuclear membrane and then subsequently often fragment, whereas the myocyte tombstone nucleus is markedly reduced in size, pale staining, and nonfragmented. When the nuclear pallor of inanotic cells is marked and the nuclear size is diminished to 2 *μ*m or less, it is assumed that the cell is nonviable. This is based upon the fact that if all of the DNA of a human nucleus was condensed, it would occupy a volume equivalent to a cube measuring 1.9 *μ*m on a side [15]. If this volume is then converted to that of a sphere to more nearly approximate the shape of a cell nucleus, it would be equivalent to a sphere with a diameter of 2.356 *μ*m. Thus, it must be assumed that cells having nuclei less than 2.356 *μ*m in diameter, as well as nuclear pallor, have lost nucleic acid material, and that such loss would be incompatible with cell survival. As we have noted in our description of inanosis in Section 3, some of the smaller inanotic nuclei measured between 1 and 2 *μ*m, although admittedly it is always difficult to exclude tangential sectioning.

Morphologic comparisons of inanosis with necrosis are more problematic than comparisons with apoptosis because necrosis is less well defined than apoptosis and also more variable in appearance. The histologic hallmarks of necrosis are usually considered to be the regional involvement of multiple contiguous cells, hypereosinophilia and swelling of the cytoplasm, variable nuclear changes including pyknosis, karyolysis, or karyorrhexis, and an accompanying inflammatory reaction because of the rupture of cell membranes with the release of cell contents. However, if the definition of necrosis is extended to include individual cell death with cytologic changes of cell shrinkage, nuclear and cytoplasmic pallor, and lack of inflammatory reaction, then inanosis would not be distinguishable from necrosis and would have to be considered as a variant of necrosis. At the least, however, there are a few features of inanosis that are not typically associated with necrosis. The degree of cell and nuclear shrinkage and pallor in inanosis is marked, the pattern of cell involvement is often mottled with viable cells between the dead cells, and there is no inflammatory reaction despite ultimate membrane degradation and release of cell contents. Necrotic cells probably do not achieve the marked degree of shrinkage seen in inanosis because there is often no preceding atrophy and because they are more rapidly eliminated by the enzymatic and phagocytic action of inflammatory cells attracted to the site by cytokines and the leakage of their cytoplasmic contents. Finally, it is of interest that autophagy, which we have shown by EM is clearly occurring in the late phase fibroid tumors, has been shown to be cytoprotective when the availability of oxygen and nutrients is poor, and in such environments is effective in inhibiting the induction of both apoptosis and necrosis [16].

In this regard, the argument might also be made that inanosis is in fact autophagic cell death. While recognizing that there is no clear answer to this question, we favor the concept that autophagy is cytoprotective to cells deprived of nutrients, and that cell death in this circumstance is more likely related to a critical lack of oxygen and essential nutrients available to the cell in its environment, rather than being secondary to a self-destructive overzealous autophagic process. If nutritionally deprived cells, struggling to survive, possess the regulatory controls to suppress apoptosis, necrosis, and apparently the release of cytokines as suggested by the lack of inflammation, then it seems likely that autophagy would also be a finely tuned and regulated survival mechanism that would not promote the demise of the cell which it is designed to protect [17, 18].

Comparative morphologic features of necrosis, apoptosis, and inanosis are summarized in Table 4.

In summary, the distinguishing features of inanosis in fibroids are the slowness of the process, the combined vascular and interstitial ischemic pathogenesis resulting from the elaboration of an extensive extracellular matrix, and the consequent cellular atrophy presumably due to nutritional and oxygen deprivation, which culminates in the formation of the hallmark cell, the myocyte tombstone. An alternative term for inanosis, combining these distinctive characteristics, is slow vasculointerstitial ischemic and atrophic cell death.

Clearly there is no evidence of phagocytosis of the inanotic cells, nor is there any reason to hypothesize enzymatic digestion by leukocytes since there is no inflammatory reaction associated with inanosis. Perhaps there is an orderly, internal as well as extracellular, disposition process for the recycling of amino acids and other important molecules from the myocyte tombstones of inanosis. This process may be viewed as a type of reclamation in which the molecular contents of the cell are reclaimed by the body and recycled. Reclamation may be mediated by both lysosomal degradation and matrix metalloproteinases. The lack of an inflammatory reaction or recruitment of macrophages seems contrary to the notion that the extrusion of cytoplasmic contents will evoke an inflammatory reaction as occurs with necrosis. It could be argued that cytokines that might be released into the dense stroma do not reach the capillaries because of the dense fibrosis; however, there seems no reason for a starving cell to release cytokines. Since it has been reported that nutritional deprivation inhibits apoptosis in cells [19], it seems plausible that starving cells might also restrict the production and release of cytokines. Evidence has also been presented that autophagy, initiated by starvation or metabolic stress, may prevent the death of cells by necrosis [20]. Finally, the electron microscopic image of shrunken, fragmented, dead myocytes, with dissolution of cell membranes and cytoplasmic contents, and extruded cell material in the adjacent stroma, without phagocytic involvement, is evidence in itself for the existence of a noninflammatory, nonphagocytic, presumably enzymatic, degradative process, which we refer to as reclamation.

From a broader standpoint, might such a mechanism as reclamation be relevant to the routine removal of effete cells from our viscera as well, since we know that most of the cells in our body have a limited lifespan and are periodically replaced? Apoptotic cells are removed by phagocytosis, and yet phagocytes with apoptotic bodies are only infrequently encountered in histologic sections of viscera. And from the standpoint of energy efficiency, why would nature develop a system that would require the energy input of a second cell (such as a phagocyte) to remove senescent cells on a regular basis?

## 5. Conclusions

We have presented morphologic studies to support our hypothesis of the contribution of collagen synthesis to both the enlargement and the eventual involution of uterine fibroids. The excessive elaboration of extracellular matrix ultimately results in hyalinization of both the smaller vessels and the interstitium, leading to reduced microvascular density, interstitial and vascular ischemia, and nutritional deprivation of the tumor myocytes. Atrophy through autophagy then develops as a survival mechanism for individual cells, until a critical point is reached when the nutritional deficits are incompatible with survival and inanotic cell death occurs. Inanosis differs from both apoptosis and necrosis in several respects, including the initiating factors, the slowness of the process, and the morphologic features. Future studies of immunohistochemical and molecular markers are needed to complement these morphologic observations and to provide additional evidence for the differentiation of inanosis from apoptosis and necrosis. Further elucidation of the process of inanosis may offer insights into the pathogenesis of cachexia in neoplastic diseases since nutritional deprivation may play a key role in each. Finally, disposition of inanotic cells seems to occur by a reclamation process of nonphagocytic, presumably enzymatic dissolution in which cellular molecules are reclaimed and recycled for utilization elsewhere.

Our proposals and our concepts are based upon our observations and our attempt to understand the biology of these tumors. Although we believe that many fibroids undergo involutional changes later in their development, we neither propose that these changes occur in all fibroids, nor that the involutional changes occurring in some fibroids result in shrinkage or disappearance of those tumors. In fact, we have shown that the later phase tumors tend to be larger on average because of the accumulated collagenous matrix, and it is the gross size of these tumors that is recognized as one of the major determinants of their morbidity.

## Figures and Tables

**Table 1:** Fibroid Phases.

Phase	Estimated collagen content	Functional status
Phase 1	No, or insignificant, collagen matrix	Proliferation of myocytes
Phase 2	<10% collagen	Proliferation of myocytes, and synthesis of collagen
Phase 3	10–50% collagen	Proliferation, synthesis of collagen, and early senescence in late Phase 3
Phase 4	>50% collagen	Involution

**Table 2:** Mitotic counts of tumors in Phases 1–4.

Phase	No. of tumors	Mitotic counts/10 HPF			
		Min	Max	Mean	S.E.
1	20	0	2.0	0.4085	0.1531
2	116	0	3.5	0.4281	0.0556
3	270	0	2.4	0.2769	0.0279
4	26	0	0.0	0.0000	0.0000

**Table 3:** Gross size of tumors in phases 1–4.

Phase	No. of tumors	No. of tumors ≥2 cm	% of tumors ≥2 cm
1	17	10	58.8
2	111	59	53.2
3	244	183	75.0
4	22	17	77.3

**Table 4:** Comparison of necrosis, apoptosis, and inanosis.

	Necrosis (ischemic)	Apoptosis	Inanosis
Basic mechanism	Energy independent	Energy-dependent	Energy independent
Speed of Process	Rapid	Rapid	Slow
Individual cell versus groups of cells	Usually groups	Individual	Individual
Typical pattern	Regional groups of cells	Individual cells	Mottled (interspersed viable cells)
Histologic hallmark	Zonal (multicellular) coagulative necrosis	Apoptotic nuclear changes; tingible body macrophages	Myocyte tombstone
Nuclear changes	Pyknosis, karyorrhexis, or karyolysis	Pyknosis and karyorrhexis	Shrinkage and pallor
Cytoplasmic volume	Cell swelling	Cell shrinkage	Cell shrinkage
Cytoplasmic shape	Variable	Rounded	Normal
Cytoplasmic tinctorial features	Hypereosinophilic	Hypereosinophilic	Pallor
Relation of cell to adjacent cells or stroma	Variable	Detaches from neighboring cells	Maintains connection initially
Inflammation	Usually	No; macrophages	No
Disposition of dead cells	Phagocytosis	Phagocytosis	Gradual dissolution (Reclamation)
Release of cytoplasmic contents	Yes	No	Yes
